# Comparison of facet joint degeneration in firefighters and hospital office workers

**DOI:** 10.1186/s40557-017-0180-1

**Published:** 2017-06-24

**Authors:** Dong Hyun Kim, Yon Soo An, Hyung Doo Kim, Kyoung Sook Jeong, Yeon-Soon Ahn, Kun-Hyung Kim, Youngki Kim, Han-Soo Song, Chul-Gab Lee, Young-Jun Kwon, Jin-Ha Yoon

**Affiliations:** 1 Department of Occupational Medicine, Dongguk University Ilsan Hospital, 29 Donggung-no, Ilsandong-gu, Goyang, 410-773 South Korea; 2 Department of Occupational and Environmental Medicine, Busan Paik Hospital, Inje University, Busan, South Korea; 3 Department of Occupational and Environmental Medicine, Busan National University Yangsan Hospital, Yangsan, South Korea; 4 Department of Occupational and Environmental Medicine, School of Medicine, Chosun University, Gwangju, South Korea; 5 Department of Occupational and Environmental Medicine, Hallym University Sacred Heaty Hospital, Anyang, South Korea; 6 Department of Preventive Medicine and Public Health, Yonsei University College of Medicine, Seoul, South Korea

**Keywords:** Firefighters, Facet joint degeneration, Low back pain, Physically demanding job, Lumbar burden

## Abstract

**Background:**

There are few published studies on the relationship between occupational lumbar load and facet joint degeneration (FJD). This cross-sectional study was conducted to evaluate the effect of physical lumbar load on FJD by comparing magnetic resonance imaging (MRI) findings of firefighters (FFs) and hospital office workers (HOWs).

**Methods:**

We randomly sampled 341 male FFs and 80 male HOWs by age stratification. A questionnaire and clinical examination, including MRI of the lumbar spine (T12-S1), were conducted. FJD was diagnosed and graded by using the classification of Pathria et al., and reclassified into two groups as follows: no FJD (grade 0) and FJD (grades 1, 2, and 3). The prevalence of FJD was analyzed according to occupational group.

**Results:**

The prevalence of FJD ranged from 31% (L1–L2) to 75% (L4–L5) in the FFs, and from 18% (L1–L2) to 69% (L4–L5) in the HOWs. After adjustment for age, body mass index, and frequency of physical exercise, the adjusted odds ratios (OR) for FJD in the FFs were significantly higher than those in the HOWs at all lumbar spinal levels, except for L3–L4 (L1–L2: OR, 2.644; 95% confidence interval [CI], 1.317–5.310; L2–L3: OR, 2.285; 95% CI, 1.304–4.006; L4–L5: OR, 1.918; 95% CI, 1.037–3.544; L5–S1: OR, 1.811; 95% CI, 1.031–3.181).

**Conclusion:**

This study shows that FFs exhibit a greater likelihood of having FJD than HOWs after controlling for other risk factors of FJD. This suggests that the physical occupational demands of FFs affect their risk of developing FJD.

## Background

Facet joint degeneration (FJD) is one of the primary causes of low back pain (LBP) [1–3]. According to the existing literature, several significant risk factors of FJD have been identified, including female sex, obesity, short stature, aging, and intense physical exercise [3–6]. Additionally, mechanical damage to the lumbar spine occurs after repetitive lumbar flexion and twisting motions [7]. For example, the prevalence of FJD in tennis athletes, who typically perform such movements, is 4 or 5 times higher than that in the age-matched general population [3, 8, 9]. In another study, strenuous physical activities were shown to be associated with a high prevalence of FJD [10]. As demonstrated in a previous study, repetitive lumbar flexion, lateral bending, and twisting induce facet joint arthropathy. However, few studies have investigated the relationship between jobs that involve a physical lumbar load and FJD [11].

Firefighters (FFs) are known to perform more physical activities in relation to their job than the general population [12]. Evaluation of job-related movements using ergonomic tools, such as the National Institute of Occupational Safety Health lifting equation, or the rapid entire body assessment, revealed that FFs frequently bend or twist their backs [7, 12]. FFs commonly perform intense physical activities involving a high load on the lumbar spine, such as fire suppression (FS), rescue operations, and emergency medical services (EMS). These activities include using heavy equipment, maintaining an improper posture in hazardous locations, carrying heavy equipment on the back, repeatedly pushing patients while transferring them, carrying patients on a stretcher, and bending their backs frequently [13–15]. Such physically demanding activities place a burden on the lumbar spine and cause degenerative lumbar diseases, such as FJD, which eventually lead to LBP among FFs [16–19]. Despite this, few studies have examined how a physically demanding job affects FJD [20].

Magnetic resonance imaging (MRI) has been found to be one of the most important diagnostic tools to identify anatomical abnormalities in the spine [21–24]. However, most MRI studies do not focus on whether degenerative changes in the lumbar spine occur in individuals with a specific job. Based on MRI, this study aimed to objectively determine whether FFs, who have a higher lumber load due to their physical activities, have a higher likelihood of developing FJD than hospital office workers (HOWs), who have a relatively lower lumber load.

## Methods

### Study subjects

#### Firefighters

In this study, Korea was divided into five areas, and five fire stations were randomly sampled for each of the five areas. We made a request to the 25fire stations sampled, and were provided with lists of FFs including information about their sex, age, and duty from each of those departments. Male FFs on the lists were stratified according to their age (those in their 20s, 30s, 40s, and 50s) and their duties at the time of the study (fire suppression, emergency medical service and rescue, and office workers). Then, they were randomly sampled in proportion to their parameters (aiming for the number of 350 FFs). Among the subjects sampled, the FFs who did not give consent for participation in this study were sampled as the second or third priority in consideration of their age and duties. Apart from those who were diagnosed by a physician with back injuries or diseases, the number of the final subjects was 341, including89 in their 20s, 96 in their 30s, 86 in their 40s, and 70 in their 50s.

#### Control group

We investigated what previous studies have done for selecting control group. Similar to our study, there was articles using “office worker” as a control. The office workers had sedentary work but were free to change posture and move around [25, 26]. So we selected hospital office workers as control group. For this study, lists of male HOWs were requested from five university hospitals located in each of the same areas as the sampled fire stations. From the lists, 20 workers each were randomly sampled according to their age group (those in their 20s, 30s, 40s, and 50s). Excluding those who were diagnosed by a physician with back injuries or diseases, the total number of subjects was 80, with 20 individuals in each age group.

### Study overview

In this study, questionnaire-based interviews, physical examination by a physician, lumbar radiography, and MRI were performed for FFs and HOWs over a 4-month period, from October 2014 to January 2015.

#### Questionnaire

The structured questionnaire consisted of questions regarding the general characteristics, occupational factors, and lifestyle factors known to affect FJD. The questionnaire was filled out by the subjects and then supplemented with interviews. The general characteristics included age (age groups: 20–29, 30–39, 40–49, and 50–59 years), height (cm), weight (kg), and body mass index (BMI).The lifestyle risk factors included smoking status (nonsmoker, ex-smoker, or current smoker), drinking status (less than 72 g alcohol intake per week or 72 g alcohol consumption per week), and frequency of physical exercise (<1 time/week, 1 or 2 times/week, or ≥3 times/week). Nonsmokers were defined as individuals who had never smoked, while ex-smokers were defined as individuals who had quit smoking >6 months prior to participating in the study. Based on the frequency of alcohol consumption per week (72 g/week), the subjects were divided into healthy drinkers and higher-risk drinkers. Frequent physical activity was defined as exercising more than 3 times/week. The occupational risk factors comprised the job duty performed for the longest duration (FFs typically perform different types of work on rotations, including fire suppression, emergency medical service and rescue, and office work) and employment duration (<5, 5–10, 10–20, and >20 years). People with lumbar spine injury or spine surgery were excluded from the study. The questionnaire survey was conducted on the presence or absence of back pain (in the past 1 year, patients complained of back pain for more than 1 day due to back pain: yes/no).

#### Physician examination

The subjects were interviewed about their medical history and physical measurements (height, weight), and then physical examinations (tenderness point in lower back, range of motion of forward backward lateral bending, sensory or motor weakness, straight leg raising test) were conducted.

#### Medical imaging tests

The protocol is pre-agreed format, imaging method and reading method. The protocol was created to score the facet joint degeneration reading. It also included disc herniation, foraminal stenosis, central canal stenosis and other spinal disease. It was essential to image and read in the same way. After a protocol was developed for simple lumbar radiography (anteroposterior, lateral, right oblique, left oblique, flexion, and extension in a standing position) and MRI, we performed medical imaging tests in accordance with the protocol, including sagittal and axial T1- and T2-weighted imaging of the spine. From the last thoracic level through the first sacral level, the MRI scans were performed on 1.5-T scanners. The slice thickness was 4 mm, and the length of the field of view ranged from 146 to 150 mm. Four radiologists and occupational environmental medicine specialists participated in the study and developed a reading paper by determining the contents to be read in simple radiographs and MR images. We determined what should be read on radiography and MRI, and developed a read sheet. By using simple radiography, we only determined the overall state in relation to the identifiable diseases and injury, such as kyphoscoliosis (scoliosis, kyphosis, and lordosis), spina bifida, spondylolysis, anterior or posterior spondylolisthesis, and fracture. After radiography, we used MRI to identify the degree of FJD (left and right), whether a slipped disk was present or absent (type of slipped disc and its direction), Pfirrmann grade (sign of a slipped disc and degeneration), the degree of central canal stenosis, and the degree of neural foraminal stenosis.

The classification method developed by Pathria et al. [27] was used to evaluate FJD. The degree of FJD on MR image was graded on a scale of 0 to 3(grade 0, normal; grade 1, degenerative changes that include joint space narrowing, cyst formation, small osteophytes without joint hypertrophy seen on axial or sagittal images; grade 2, joint hypertrophy and large osteophytes without fusion; and grade 3, bony fusion of the joint [28]). In the case of disagreement betweenthe readers about the grade, the higher grade was selected. To evaluate the degree of degeneration of the facet joints, each MR image was independently analyzed by two radiologists. Each radiologist read half of total MR images of subjects. A total four radiologists participated in reading MR images. The gamma values were compared in all spine segments in either left or right between the two radiologists. Kruskal’s gamma is a measure of rank correlation. It measures the strength of association of the cross table data. Value range from −1(100% negative association) to +1(100% positive association) [29]. The agreement among readings was a gamma value of 0.458–0.77.

#### Outcome

With respect to the primary outcome, this study aimed to determine the odds ratio of occurrence of FJD at each lumbar level of the FFs, with the HOWs serving as the control group. The occurrence of FJD was defined if Pathria facet joint degeneration grade is 1, 2 or 3 in either right or left side at spine segment.

### Statistical analyses

To compare the risk factors for FJD (age, height, weight, body mass index, frequent exercise, working period, back pain experience, smoking, drinking) between FFs and HOWs, the t-test and chi-squared test were used. The chi-squared test, Mann-Whitney test, and ANOVA were used to compare FJD between age groups.

The odds ratio (OR) with 95% confidence interval (95% CI) of FJD among FFs as compared to HOWs was evaluated by binary logistic regression after controlling for age, BMI, smoking, and physical exercise.

To compare the risk factors for FJD (age, BMI, and frequent physical exercise) between the FFs and HOWs, we chose FJD as a dependent variable and age, BMI, and frequent exercise as independent variables in the *t*test, chi-square test, Mann-Whitney test, ANOVA, and multiple logistic regression analysis. With the HOWs as the control group, the FJD of the FFs was adjusted forage, BMI, smoking, and frequent physical exercise, and then compared for the odds ratio at a 95% confidence interval (CI). Statistical significance was determined as a *p*value of <0.05. R version 3.1.0 with moonBook packages was used for all the data analyses.

## Results

### General characteristics of the participants

The general characteristics of the participants in this study are shown in Table 1. There was no statistically significant difference in the age, body mass index, frequency of exercise, working period, or alcohol consumption between the two groups. The proportion of FFs who reported that they felt back pain for more than 1 day was significantly higher than that of HOWs (68.1% versus 55.0%, *p*=0.027).

**Table 1:** General characteristics of study participants

Variables		Firefighters(N = 341)		Office workers(N = 80)		*p*-value
		*n*	*%*	*n*	*%*	
Age (years)	20–29	89	26.0	20	25	0.832
	30–39	96	28.4	20	25	
	40–49	86	25.1	20	25	
	50–59	70	20.5	20	25	
Height (cm)	Mean ± SD	174.0 ± 5.12		174.7 ± 10.43		0.391
Weight (kg)	Mean ± SD	74.0 ± 8.11		74.2 ± 10.11		0.376
BMI	Mean ± SD	24.4 ± 2.49		25.0 ± 3.12		0.137
Physical exercise(times/week)	<1	112	32.9	23	28.8	0.703
	1~2	113	33.1	30	37.5	
	≥3	116	34.0	27	33.7	
Duration of employment(years)	<5	112	32.8	21	26.2	0.771
	5–9	48	14.1	14	17.5	
	10–14	32	9.4	8	10.0	
	15–19	51	15.0	11	13.8	
	≥20	98	28.7	26	32.5	
Alcohol consumption	Healthy drinker	93	27.2	15	18.8	0.116
	High risk drinker	248	72.8	65	81.2	
Smoking status	Non-smoker	119	34.9	31	38.7	0.397
	Ex-smoker	121	35.5	22	27.5	
	Current smoker	101	29.6	27	33.8	
Episode of back pain	No	109	31.9	36	45.0	0.027
	Yes	232	68.1	44	55.0	

*SD* standard deviation, *BMI* body mass index, Episode of back pain: 1 day with complaint from the lower back during the previous 1 year; Physical exercise: the number of exercises for more than 30 minutes per week; Healthy drinker: drink less than 72 grams of alcohol per week; High risk drinker: drink more than 72 grams of alcohol per week

### Comparison of the prevalence of FJD in FFs and HOWs

There was a significant difference in the prevalence of FJD between FFs and HOWs at the L1–2 level (30.5%vs 17.5%, *p*=0.018) and the L2–3 level (51.0% versus 38.8%, *p*= 0.048).The difference in the prevalence of FJD between two groups at the L3–4, L4–5, and L5-S1 levels was not statistically significant (Table 2).

**Table 2:** Prevalence of facet joint degeneration (FJD) stratified by occupation in each spine segments

Segment	Firefighters		Office workers		*p*-value
	*n*	*%*	*n*	*%*	
L12	105	30.5	14	17.5	0.018
L23	174	51.0	31	38.8	0.048
L34	200	58.7	44	55.0	0.585
L45	257	75.4	55	68.8	0.225
L5S1	217	63.6	42	52.5	0.066

*L12* lumbar spine levels 1–2, *L23* lumbar spine levels 2–3, *L34* lumbar spine levels 3–4, *L45* lumbar spine levels 4–5, *L5S1* lumbar spine level 5 and sacral spine level 1, *FJD* FJD was defined if Pathria facet joint degeneration grade is 1, 2 or 3 in either right or left side

As compared to the HOWs, the odds ratios of FJD prevalence among the FFs were not significant. The odds ratios adjusted for age, BMI, smoking, and frequent exercise, all of which are known to be risk factors for FJD. Except for FJD at the L3–4 level, the odds ratios of FJD at the L1–2, L2–3, L4–5, and L5-S1 levels were statistically significantly higher in the FFs than in the HOWs (Table 3).

**Table 3:** Odds ratio for facet joint degeneration (FJD) at various spine levels, measuring risk in emergency responders compared to risk in hospital office workers

Segment	Unadjusted odds ratio (95% CI)	*p*-value	Adjusted odds ratio (95% CI)	*p*-value
L12	1.78 (0.97–3.30)	0.071	2.64 (1.32–5.31)	0.006
L23	1.49 (0.91–2.41)	0.125	2.29 (1.30–4.01)	0.004
L34	1.08 (0.67–1.74)	0.778	1.41 (0.81–2.46)	0.228
L45	1.27 (0.76–2.11)	0.384	1.92 (1.04–3.54)	0.038
L5S1	1.41 (0.87–2.26)	0.171	1.81 (1.03–3.18)	0.039

*CI* confidence interval, *L12* lumbar spine levels 1–2, *L23* lumbar spine levels 2–3, *L34* lumbar spine level 3–4, *L45* lumbar spine levels 4–5, *L5S1* lumbar spine level 5 and sacral spine level 1, *FJD* FJD was defined if Pathria facet joint degeneration grade is 1, 2 or 3 in either right or left side; Adjusted factors: age, body mass index, frequency of physical exercise

## Discussion and conclusions

We analyzed and compared the prevalence of FJD in FFs and HOWs according lumbar level. The results showed that, the FJD prevalence was higher among the FFs than among the HOWs at all lumbar levels, except for the L3–4 level, and this difference were significant. These findings were noted after adjustment for age, BMI, and frequent physical exercise, which are potential risk factors for FJD; moreover, the odds ratio of FJD was 1.81 to 2.64 times higher for the FFs than for the HOWs.

There is the suspected reason of indifference in L3-L4 level between two groups. There are two reasons for these findings. First, there would be a significant difference affecting lower lumbar segment between FF and HOW’s working environment. Second, the main difference between FFs and office workers (OWs) may be both manual material handling (lifting) and lumbar bending or twisting [7], which might affects upper lumbar levels more than lower lumbar levels. Considering that FJD at the lower lumbar levels is common in both groups (FFs and OWs), it is reasonable that a remarkable difference exists in the upper lumbar levels. L3–4 is neither the upper part nor the lower part. L3–4 load, twisting motion was not enough to cause the FJD difference between two groups. However, data that support these assumptions are still lacking, and further study is needed.

Risk factors known to cause FJD include age, sex, race, anatomically abnormal lumbar structure (e.g., scoliosis), individual genetic predisposition, overweight, long-time sedentary posture, occupation, nutrition, and lifestyle habits [3, 30, 31].

According to Panjabi, FJD exacerbated as it became closer to the bottom of the lumbar, and L45 was worse than L5S1 [31, 32]. In this study, both the FFs and OWs showed that as FJD became closer to the bottom of the lumbar, its prevalence increased. The results revealed that degeneration atL45 was more advanced than at L5S1, which were consistent with the results of the aforementioned study. This could be because as it went down near the bottom, the height of the disk lowered and the caudal segment motion of the lumbar increased, placing more loads, which was heaviest at L45.

The authors of a previous study [30] argued that FJD occurs when the load in facet joints increases due to a lowering disk height and spinal segmental instability arising from vertebral disk degeneration. However, other researchers suggested a weak correlation between disk degeneration and FJD [33–35]. Nevertheless, greater physical load on facet joints means that nearby disks, ligaments, muscles, and other supporting systems weaken, or as segmental instability increases in the spine, it applies more mechanical load to facet joints. Even without medical history of disk diseases or abnormalities in ligaments and muscles near the lumbar, repeated motion could increase segmental instability in the spine. We think field activity of FFs represents the stress on lumbar spine. We believe that an ergonomic study will be needed in the future to investigate whether repeated labor increases spinal segmental instability and accordingly places greater physical lumbar load in facet joints.

This study has some limitations. First, it is a cross-sectional study and hence, we could not track change of individual FFs and HOWs. Second, the FF group might have had a healthy worker effect as that noted in FFs involved in the World Trade Center collapse [36]. FFs are selected people with certain level of performance. If FFs are injured, they could change task or quit their job. For this reason, the differences between the two groups in this study might have been diluted. Third, we did not quantify lumbar burden for each duty and did not determine each of their correlations with FJD. Fourth, we did not evaluate the objective evidence of lumbar loading. Fifth, we did not include the duration of job employment as an adjusting factor, although working duration may be an important consideration when comparing firefighters and hospital office workers. Lastly There was no statistically significant difference in simple prevalence between the two groups because the number of HOW was small. If there were a large number of HOW’s, there would have been a difference because of the increase in statistical power.

This study has several advantages as compared to previous studies. First, the study was well designed, and used a stratified random sampling method. Second, while the existing studies examined differences in lumbar diseases among occupations, mainly with respect to LBP prevalence, this study evaluated lumbar changes using MRI, an objective diagnostic tool. FJD differences between the two groups were clearly observed by MRI. This is in contrast to previous studies that explained differences in occupational burden based on LBP alone [37–42], and to a study that used MRI and compared nurses according to each duty to determine the presence or absence of LBP or severe disk degeneration [43]. This study selected FFs with a high lumbar load and HOWs, who were believed to have relatively lower lumbar loads, and demonstrated that occupational lumbar load could lead to FJD. Third, the study included a sufficient number of subjects, which was >400; however, to the author’s knowledge, other study that looked at the relationship between occupation and lumbar degeneration through MRI were 109 cases [43]. Fourth, by matching stratified random samples with the control group, we reduced the selection bias in this study. Fifth, this study did not include women who were at risk of developing FJD while adjusting for risk factors such as age, BMI, and frequent physical exercise to determine the odds ratio, thereby minimizing the effect of confounding factors that could affect the results of this study. Lastly, this study is the first to address FJD in FFs and is one of the rare studies to examine correlations between occupational lumbar load and FJD. This study is meaningful in that firefighting as an occupation was found to have a significant correlation with the development of FJD.

In conclusion, the development of FJD has significant relationship with occupational lumbar load. This study demonstrated that the prevalence of FJD was higher among FFs than among HOWs. This shows a clear correlation between FFs’ occupational lumbar load and the risk of developing FJD. However, additional studies are needed to investigate correlations between activities that confer lumbar burden and lumbar degenerative diseases, occupational lumbar burden (quantification), correlations between occupational lumbar load and FJD in occupations other than firefighting, and correlations between degenerative changes and LBP.

## Abbreviation

BMI: Body mass index
CI: Confidence interval
EMS: Emergency medicalservices
FFs: Firefighters
FJD: Facet joint degeneration
FS: Fire suppression
HOWs: Hospital office workers
LBP: Low back pain
MRI: Magnetic resonance imaging
OR: Odds ratios
